# Knowledge, attitude and practice towards malaria among symptomatic patients attending Tumbi Referral Hospital: A cross-sectional study

**DOI:** 10.1371/journal.pone.0220501

**Published:** 2019-08-05

**Authors:** David Zadock Munisi, Azan A. Nyundo, Bonaventura C. Mpondo

**Affiliations:** 1 Department of Parasitology and Entomology, School of Medicine and Dentistry, College of Health Sciences, University of Dodoma, Dodoma, Tanzania; 2 Department of Psychiatry, School of Medicine and Dentistry, College of Health Sciences, University of Dodoma, Dodoma, Tanzania; 3 Department of Internal Medicine, School of Medicine and Dentistry, College of Health Sciences, University of Dodoma, Dodoma, Tanzania; Instituto Rene Rachou, BRAZIL

## Abstract

**Background:**

Despite significant improvement in prevention and control over the past decades malaria remains a significant public health concern in Tanzania with 93% of the population being at risk. To prevent malaria infection and promote malaria free zones, understanding the community’s knowledge, attitudes, and practices toward malaria control are essential. This study therefore aimed at determining the levels of understanding, and attitudes, as well as socio-cultural aspects of malaria prevention and treatment-seeking behaviours among suspected malaria patients.

**Methods:**

This study was a hospital based cross-sectional study, in which patients attending Tumbi Referral Hospital with symptoms and signs that warrant inclusion of suspicion of malaria, were recruited. We used a pre-tested semi-structured questionnaire to collect participants’ demographic characteristics, as well as information on their knowledge, attitudes, and practices towards malaria infection. Data were analysed using Stata Version 12.1.

**Results:**

We enrolled a total of 295 respondents of which 179 (60.68%) were females. Participants’ ages ranged from 1–91 years, with a mean of 31.4 years. Seventy-nine (26.8%) patients reported having malaria in the previous 28 days, with 57 (72.2%) being laboratory confirmed. Only 52 (65.8%) individuals reported taking prescribed medications for malaria. A total of 277 (93.90%) were aware of malaria, and 264 (95.31%) knew that it is transmitted by mosquito. Nearly all participants (263, 94.95%), identified sleeping under bed nets to be protective against malaria. About half of the respondents either agreed 63 (22.74%) or strongly agreed 62 (22.38%) that malaria can be transmitted like the common cold. Self-reported mosquito net use was 88.09% (244).

**Conclusion:**

Despite the endemicity of malaria in our study site, patients had adequate knowledge, encouraging attitudes, and good practices related to malaria prevention and control.

## Introduction

Malaria is an infectious vector-borne disease caused by four common species of protozoan parasites of the genus Plasmodium: *Plasmodium falciparum*, *Plasmodium vivax*, *Plasmodium ovale* and *Plasmodium malaria* [1]. A global reduction in investment in malaria research and control in 2016 conversely led to an increase from 2015 of 211 million malaria cases to 216 million in 2016 [2]. Of all global malaria cases, 90% occur in the World Health Organization’s Africa Region with 15 sub-Saharan countries carrying 80% of the global burden of malaria [2]. *Plasmodium falciparum* accounts for about 99% of all the malaria cases [2]. Apart from being the major cause of morbidity and mortality, malaria programs account for over US$12 billion annually in African nations [3].

Despite significant improvement in prevention and control for the past decades, malaria remains a significant public health concern in Tanzania with 93% of the population at risk for malaria infection [4]. It remains among the leading national causes of morbidity and mortality especially in children under five years and pregnant women whereby malaria dominates outpatients, inpatients and admissions of under five year children at health facilities [4].

Malaria is a treatable and preventable disease requiring well-organized and delivered intervention programs. Tanzania, along other sub-Saharan African countries, adopted the “President’s Malaria Initiative”, which was launched in 2005 to reduce malaria-related mortality by 50% [5]. Malaria reduction is targeted through a rapid scale-up of four proven and highly effective malaria prevention and treatment measures: insecticide-treated mosquito nets (ITNs); indoor residual spraying (IRS); accurate diagnosis and prompt treatment with artemisinin-based combination therapies (ACTs); and intermittent preventive treatment of pregnant women (IPTp) [5]. The Tanzanian National Malaria Control Program Strategic plan for 2015–2020 calls for the reduction of malaria morbidity and mortality by 80% by 2020, reduction of malaria prevalence from 5% in 2016 to 1% by 2020, and increased proportion of women receiving two or more doses of SP during their pregnancy from 32% in 2012 to 80% by 2016 [5]. To attain the expected results and sustain the expected gains, strengthening of the health system and capacity building is crucial [5].

To achieve the above national goals, community knowledge, attitudes and practices towards malaria are essential in preventing malaria infection and promoting malaria free zones [1]. The success of malaria control efforts is highly dependent on the level of understanding, attitudes, and socio-cultural aspects of malaria prevention and treatment-seeking behaviours in the community [6].

Previous African-based studies regarding knowledge, attitude and practices about malaria and its control concluded that unsatisfactory practices and misconceptions towards malaria persist [7]. Therefore, we must improve our knowledge of community beliefs and practices respecting malaria in order to engage and embed them in surveillance and control activities [8]. Failure to consider the communities’ knowledge, attitudes, and practices towards malaria will impact the achievement of successful sustainable control programs [9], as studies on malaria control programs have demonstrated direct interaction with community plays a crucial role in dealing with malaria problems [10]. Addressing community knowledge, attitudes, and perceptions can be of critical importance towards developing malaria control strategies [8], understanding who knows what about malaria and malaria prevention, who adopted malaria prevention and mosquito avoidance, who is at risk for malaria infection are all precursors to successful implementation and sustainability of malaria control efforts [11]. There remains scarcity of studies regarding knowledge, attitudes, and practices in Tanzania. As there is a scarcity of studies on this important topic, our study aimed to determine knowledge, attitude and practices towards malaria among symptomatic patients attending Tumbi Referral Hospital in the Coastal Region of Tanzania.

## Methodology

### Study setting

The study was conducted at Tumbi Referral hospital located in the Coastal Region, which is about 45 kilometers from Dar-es-Salaam, which was previously the central administrative and most populous city in the country. Tanzania’s Coastal Region has one of high endemic rates for malaria throughout the year, with highest transmission intensity during the rainy seasons. The study was conducted during and just after the rain season from December, 2017 to May, 2018.

### Study design

This study was a hospital based cross sectional study.

#### Study population

The study involved all patients attending at the hospital with symptoms and signs that warrant inclusion of malaria (suspected malaria cases of all ages).

#### Sample size

The minimum number of study subjects was estimated by using a sample size formula by Kish and Leslie for cross-sectional studies [12]:

$$=\frac{z\alpha^{2}p(1-p)}{\delta^{2}}$$

Where Zα = Standard normal deviate at 95% confidence interval corresponding to 1.96; P = Assumed true population prevalence of *Plasmodium falciparum* malaria, assumed to be 30%, from a previous study done in Kibaha District, Coastal Region [13]; δ = Absolute error between the estimated and true population prevalence of *Plasmodium falciparum* malaria of 5% or 0.05; and n = estimated sample size.

$$=\frac{1.96^{2}\times 0.3(1-0.3)}{0.5^{2}}$$

yields a minimum sample size of 323 study participants for this study.

#### Sampling procedure

Systematic sampling procedure was used to recruit study participants. A sampling interval of five (5) patients was used to select participants from patients with signs and symptoms consistent with malaria and who were referred to the outpatient laboratory for blood smears. Questionnaires were collected between December 2017 to May 2018.

### Data collection

#### Assessment of socio-demographic information and risk factors

A pre-tested Kiswahili translated semi-structured questionnaire was used to capture demographic characteristics (age, sex, socio-economic status) of the study participants as well as gather information on their knowledge, attitudes, and practices towards malaria infection. The questionnaire probed for the participants’ knowledge of malaria including its transmission and symptoms; prevention and control measures/practices and use of ITNs. The questionnaire was initially developed in English and then translated to Kiswahili and back-translated by a different person who was blinded to the original questionnaire. For children who could not express themselves and comprehend the questions, the parent/caregiver was interviewed instead.

#### Data analysis

The collected data were entered into a database using EpiData Version 3.1. Data analysis was done using STATA Version 12.1 (Stata Corp, Texas, USA). Descriptive statistics, including percentages and mean values, were used to visualize the data, augmented with graphs drawn using Microsoft Excel program.

#### Ethical statement

The study was approved by the University of Dodoma, Institutional Research Review Committee. Before enrolment into the study, the objectives of the study and the study procedures to be followed were explained to the patients. Written informed consent was sought from all study participants who were informed of their right to refuse to participate and/orto withdraw from the study at any time. Assent was sought from children, who were also informed of their right to refuse to participate and to withdraw at any time during the study, and their parents or guardians provided a signed informed consent form.

## Results

### Socio-demographic characteristics of study participants

We enrolled a total of 295 respondents, with the majority being females [179 (60.68%)]. The age of the respondents ranged from 1–91 years, with a mean age of 31.4 years. The majority of respondents [130 (44.07%)] had secondary education with only 25(8.47%) reporting to no formal education. The main economic activities by study respondents were agriculture [75(25.42%)], formal employment [66(22.37%)], and trading [64(21.69%)] (Table 1).

**Table 1:** Socio-demographic characteristics.

Characteristic	Frequency(%)
**Sex (n = 295)**	
Male	116 (39.32)
Female	179 (60.68)
**Age (years) (n = 295)**	
1–20	91 (30.85)
21–40	117 (39.66)
41 and above	87 (29.49)
**Pregnant (n = 179)**	
Yes	16 (8.94)
No	163 (91.06)
**Level of education (n = 295)**	
No formal education	25 (8.47)
Primary	108 (36.61)
Secondary	130 (44.07)
Tertiary	32 (10.85)
**Main economic activity (n = 295)**	
Formal employment	66 (22.37)
Casual labour	19 (6.44)
Trading	64 (21.69)
Agriculture	75 (25.42)
Crafts	26 (8.81)
Support from relative	45 (15.25)

### Clinical characteristics of study participants

Out of the 295 study participants, 79 (26.8%) reported having malaria within the previous 28 days of whom 57 (72.2%) was laboratory confirmed. All 79 participants reported using anti-malarial drugs but only 52 (65.8%) reported taking prescribed medications.

### Respondents’ knowledge of malaria transmission and symptoms

Respondents were asked if they have ever heard of malaria to which 277(93.90%) responded positively. The most common sources of malaria related information were television 186 (67.15%), radio 155 (55.96%), family members 154 (55.60%) and health facilities 142 (51.26%). Most study participants [264 (95.31%)] were aware that malaria is transmitted via a bite by mosquito infected with malaria; however, 8 (2.89%), 3 (1.08%) and 2 (0.72%) reported other means of transmittance such as close contact with a malaria patient, eating lots of mangoes, and drinking contaminated water respectively. The three most commonly mentioned symptoms of malaria included elevated temperature [253 (91.34%)], headache [215 (77.62%)], and vomiting [210 (75.81%)]. The study further assessed the knowledge of participants on malaria prevention and control measures. Out of 277 participants who responded to this question, 263 (94.95%) identified sleeping ITNsto be protective against malaria. However, knowledge on other preventive measures was low; only 107 (38.6%) identified wearing long sleeved clothes to be protective against malaria; 146 (52.7%) identified spraying of insecticides; and 116 (41.9%) identified trimming of bushes to be protective against malaria (Table 2).

**Table 2:** Knowledge on malaria, transmission, symptoms, prevention and control measures.

Characteristic	Frequency (%)
**Heard of malaria (n = 295)**	
Yes	277 (93.90)
No	18 (6.10)
**Source of information (n = 277)**	
Family member	154 (55.60)
Neighbour	104 (37.55)
Radio	155 (55.96)
Television	186 (67.15)
Newspaper	74 (26.71)
Poster	38 (13.72)
School	121 (43.68)
Church	23 (8.30)
Health facility	142 (51.26)
Drugs shop	15 (5.42)
**How can malaria be transmitted to man (n = 277)**	
Bite by mosquito infected with malaria	264 (95.31)
Coming into close contact with a malaria patient	8 (2.89)
Eating lots of mangoes	3 (1.08)
Drinking contaminated water	2 (0.72)
**The following are signs/symptoms of malaria (n = 277)**	
Elevated temperature	253 (91.34
General body weakness	133 (48.01)
Vomiting	210 (75.81)
Sweating	108 (38.99)
Headache	215 (77.62)
Chills	97 (35.02)
Dizziness	108 (38.99)
I don’t know the signs and symptoms of malaria	8 (2.89)
**The following can be used to prevent/control malaria**	
**Sleeping under bed nets (n = 277)**	
Yes	263 (94.95)
No	14 (5.05)
**Wearing long sleeved clothing (n = 277)**	
Yes	107 (38.63)
No	170 (61.37)
**Spraying insecticides (n = 277)**	
Yes	146 (52.71)
No	131 (47.29)
**Trimming bushes around the house (n = 277)**	
Yes	116 (41.88)
No	161 (58.12)
**Cleaning dark corners in the house (n = 277)**	
Yes	110 (39.71)
No	167 (60.29)
**I don’t know (n = 277)**	
Yes	7 (2.53)
No	270 (97.47)

### Need for more information about malaria

We asked participants whether they felt that they had enough information about malaria, yield a majority response of no by 41%, and with 37% responding yes. In most responses, malaria prevention (51.75%) and control (35.09%) were areas indicated as needing more information.

### Participants’ attitudes towards malaria

In this study we assessed participants’ attitudes towards malaria. Most respondents agreed 105 (37.91%) or strongly agreed 146 (52.71%) that malaria is a serious and life-threatening disease. Close to a half of the respondents, either agreed 63 (22.74%) or strongly agreed 62 (22.38%) that malaria can be transmitted from one person to another like the common cold. More than three-quarters of the subjects either agreed 124 (44.77%) or strongly agreed 90 (32.49%) that the best self-prevention was to avoid getting mosquito bites. About 90% (247/277) of respondents said they were sure that anyone could get malaria. More than 85% (237/277) of the respondents agreed or strongly agreed that sleeping under an ITN prevents contracting malaria. Around 27% (75/277) of the respondents said they could treat themselves when they get malaria, whilst 19% (52/277) said one can recover spontaneously from malaria. Most respondents [79.06% (219/277)] agreed or strongly agreed that it is dangerous when malaria medicine is not taken completely. Of the respondents 48.73% (135/277) either agreed or strongly agreed that they can buy anti-malaria drugs from the medicine shop/pharmacy to treat themselves when they get malaria. The majority [88.08% (244/277)] of respondents thought they should go to the health centre/clinic to confirm malaria via a blood test (Table 3).

**Table 3:** Participants’ attitudes towards malaria.

Variable	Strongly Disagree n (%)	Disagree n (%)	Agree n (%)	Strongly Agree n (%)
Malaria is a serious and life-threatening disease	13 (4.69)	13 (4.69)	105 (37.91)	146 (52.71)
Malaria can be transmitted from one person to another like the common cold	44 (15.88)	108 (38.99)	63 (22.74)	62 (22.38)
The best way to prevent myself getting Malaria is to avoid getting mosquito bites	22 (7.94)	41 (14.80)	124 (44.77)	90 (32.49)
I am sure that anyone can get malaria	6 (2.17)	24 (8.66)	127 (45.85)	120 (43.32)
Sleeping under a mosquito net can prevent myself getting malaria	6 (2.17)	34 (12.27)	139 (50.18)	98 (35.38)
I can treat myself if I get malaria	54 (19.49)	148 (53.43)	61 (22.02)	14 (5.05)
Only children and pregnant women are at risk of malaria	43 (15.52)	120 (43.32)	89 (32.13)	25 (9.03)
One can recover spontaneously from malaria	77 (27.80)	148 (53.43)	40 (14.44)	12 (4.33)
If someone has malaria, people should avoid having close contact with him/her	88 (31.77)	126 (45.49)	50 (18.05)	13 (4.69)
I might be at a greater risk of getting malaria if I work and sleep overnight in the garden or forest	37 (13.36)	62 (22.38)	142 (51.26)	36 (13.00)
It is dangerous when malaria medicine is not taken completely	22 (7.94)	36 (13.00)	152 (54.87)	67 (24.19)
I can buy anti-malaria drugs from the drug shop/pharmacy to treat myself when I get malaria	37 (13.36)	105 (37.91)	112 (40.43)	23 (8.30)
I think that I should go to the health centre/clinic to have my blood tested as soon as I suspect that I am suffering from malaria	17 (6.14)	16 (5.78)	182 (65.70)	62 (22.38)

### Malaria preventive measures used by study participants

We assessed the malaria preventive measures used by study respondents. Respondents were inquired on possession and source of ITN used at home. Most (84%) respondents possessed an ITN at home and most (52.43%) respondents reported to buy ITNs at shops, while 39.32% reported obtaining the ITN free of charge from campaigns. The majority of respondents [244 (88.09%)] were using mosquito nets to protect themselves against malaria. Other malaria prevention measures reported by study respondents included: use of mosquito repellents [107 (38.63%)], closing windows [94 (33.94%)], and using a mosquito coil [65 (23.47%)] (Table 4).

**Table 4:** Malaria preventive measures reported by study participants.

Malaria preventive measure	Frequency (%)
**Own an ITN at home (n = 245)**	
Yes	206 (84.00)
No	39 (16.00)
**Source of ITN (n = 206)**	
Buying at shops	108 (52.43)
Free of charge from campaigns	81 (39.32)
Subsidized price from health facility	17 (8.25)
**Use mosquito nets** (n = 277)	
Yes	244 (88.09)
No	33 (11.91)
**Use mosquito repellents** (n = 277)	
Yes	107 (38.63)
No	170 (61.37)
**Use mosquito coil** (n = 277)	
Yes	65 (23.47)
No	212 (76.53)
**Use mosquito spray** (n = 277)	
Yes	6 (2.17)
No	271 (97.83)
**Close windows** (n = 277)	
Yes	94 (33.94)
No	183 (66.06)
**Have window screen** (n = 277)	
Yes	14 (5.05)
No	263 (94.95)

### Malaria prevention practices used by the study respondents

This study further assessed malaria prevention practices by study respondents. It was found that the majority of participants reported to always use bed nets themselves [214 (76.2%)], together with their family members [185 (65.8%)], while the remainder reported sometimes using the ITNs. Furthermore, the majority of participants reported sometimes using mosquito repellents (55.1%) or sprays (58.0%), clearing bushes near to homes (71.89%), removing stagnant water (65.84%) and visiting health facility when falling sick (55.9%) (Table 5).

**Table 5:** Participant’s malaria prevention practices (n = 281).

Variable	Always (n,%)	Sometimes (n,%)	Never (n,%)
How often do you sleep in a mosquito net?	214 (76.16)	63 (22.42)	4 (1.42)
How often do other members of the household sleep in mosquito nets?	185 (65.84)	93 (33.10)	3 (1.07)
How often do you check for holes/repair mosquito nets?	63 (22.42)	180 (64.06)	38 (13.52)
How often do you use mosquito repellent coils in your house?	35 (12.46)	155 (55.16)	91 (32.38)
How often do you use anti-mosquito spray in your house?	35 (12.46)	163 (58.01)	83 (29.54)
How often do you clean/cut bushes around your house?	63 (22.42)	202 (71.89)	16 (5.69)
How often do you clean stagnant water near your house?	62 (22.06)	185 (65.84)	34 (12.10)
How often do you visit the health centre when you fall sick?	107 (38.08)	157 (55.87)	17 (6.05)

## Discussion

This study aimed at assessing the level of knowledge, attitudes, and practices towards malaria among symptomatic patients attending Tumbi Referral Hospital.Overall, we found adequate knowledge, encouraging attitudes and good practice on malaria prevention and control.

In this study, we found that amongs the 79 participants who reported having malaria and using anti-malarial medications in the past 1-month, approximately 28% did not confirm the diagnosis through laboratory examination, a practice which is discouraged by the World Health Organization. It recommends that all individuals with symptoms and signs suggestive of malaria should have a confirmatory parasitological test, either via microscopy or Rapid Diagnostic Test (RDT) [14]. Symptomatic management of malaria has been a common practice in Tanzania [15], as in other endemic settings [16]. This study revealed that about 34% of individuals who used anti-malarials in the past month were using drugs not prescribed to them. The use of self-administered, over-the-counter medications has been reported elsewhere in East Africa East Africa [17] and has a documented history [18].

This study has found participants’ awareness on malaria is high, which was similarly reported in another study in Tanzania [19]. However, awareness alone is insufficient as one must understand the causes and modes of transmission in order to achieve disease control [20]. Individuals mentioned that the most common source of information about malaria was television followed by radio and family member signifying these as strategic channels through which to deliver malaria-related information to the community. These findings are in line with other studies done in malaria endemic settings which report that over 90% of individuals in such settings are aware of malaria ad that television, radio and family members are the most frequently mentioned source of malaria information sources [21,22]. One caution in terms of generalizability is that this study occurred in a peri-urban area with high television penetration which is not necessarily representative of the country’s more rural demography. Similar to other studies in malaria endemic settings, we found that about 95% of participants knew that malaria is transmitted by a mosquito bite [21,22]. However, our findings differed with a study done in Zanzibar in which the majority of participants could not associate malaria and mosquito bites. This variance may be attributed to the low endemicity of malaria in Zanzibar compared to mainland Tanzania. In terms of symptoms, over 90% of participants correctly identified fever while over 75% of the individuals mentioned headache as a symptom. Similar findings were obtained in studies done in other malaria endemic settings respecting mosquite bite as vector and the same presenting symptoms [7, 21,23,24].

In our study, about 95% of individuals mentioned sleeping under ITNs as a means for malaria prevention and control, which is similarly reflected in previous studies [25,26]. As the participants reported sharing the ITNs with family members, this was evidence of their knowledge of the protective use of ITNs. Tanzania through the Global Fund has been conducting ITN distribution campaigns with the aim of universal coverage. In our study, the majority of the participants (> 80%) reported hearing about ITN and/or owning an ITN, which is comparable to other studies done in East Africa [26,27]. More than half of the participants reported radio, television and family member as the source for ITN knowledge. Despite the net distribution campaign, less than 40% of participants reported to have obtained the nets from the distribution campaign making this an area for improvement and optimization which has been reported previously [28,29]. Free distribution, despite improving coverage, has been shown to reduce inequity in ownership and use as well [29]. Despite knowledge on ITNs as protective, a significant number of participants did not know that wearing long sleeved clothes, clearing of the bushes close to the houses, and mosquito sprays can prevent transmission of malaria. Knowledge translated into practice was seen in their self-reported clearing of bushes and clearing stagnant water, while very few reported using mosquito repellents/sprays. Similar findings were reported in a study done in Nigeria where the use of repellents and spraying was low; however wearing of long sleeved shirts was high in the Nigerian study than our study [21]. This knowledge to practice aspect must be addressed in future campaigns to further reduce malaria transmission in the country.

## Limitations

We acknowledge that not having recruited the required sample size might have compromised the power of our study. Furthermore, we acknowledge that the study was done in a peri-urban area which may have impacted the level of knowledge that is reported by this study (due to higher education levels, sources of income, and access to health information). Finally, we acknowledge that the hospital-based study population may not be representative of the general population, and limits our inference to febrile patients attending Tumbi Referral Hospital.

## Conclusion and recommendations

In our malaria endemic study site, participants had adequate knowledge, encouraging attitudes, and good practices on malaria prevention and control. However, a few misconceptions on malaria disease, its transmission, and prevention persist as reported in this study, which ideally should be addressed and clarified before launching a comprehensive malaria control program in the area. Using findings from this study, messaging to clarify and engage people in future malaria programs in this area should be done through television, radio or engaging family members.

## Supporting information

S1 Data Knowledge attitude and practices on malaria dataset. (DTA)
